# Plasmid-Cured *Chlamydia caviae* Activates TLR2-Dependent Signaling and Retains Virulence in the Guinea Pig Model of Genital Tract Infection

**DOI:** 10.1371/journal.pone.0030747

**Published:** 2012-01-24

**Authors:** Lauren C. Frazer, Toni Darville, Kumar Chandra-Kuntal, Charles W. Andrews, Matthew Zurenski, Margaret Mintus, Yasser M. AbdelRahman, Robert J. Belland, Robin R. Ingalls, Catherine M. O'Connell

**Affiliations:** 1 Department of Pediatrics, Children's Hospital of Pittsburgh of UPMC, Pittsburgh, Pennsylvania, United States of America; 2 Milstead Pathology Group, Conyers, Georgia, United States of America; 3 Department of Microbiology, Immunology, and Biochemistry, University of Tennessee Health Science Center, Memphis, Tennessee, United States of America; 4 Department of Microbiology and Immunology, Faculty of Pharmacy, Cairo University, Cairo, Egypt; 5 Department of Medicine, Boston University School of Medicine, Boston, Massachusetts, United States of America; University of California Los Angeles, United States of America

## Abstract

Loss of the conserved “cryptic” plasmid from *C. trachomatis* and *C. muridarum* is pleiotropic, resulting in reduced innate inflammatory activation via TLR2, glycogen accumulation and infectivity. The more genetically distant *C. caviae* GPIC is a natural pathogen of guinea pigs and induces upper genital tract pathology when inoculated intravaginally, modeling human disease. To examine the contribution of pCpGP1 to *C. caviae* pathogenesis, a cured derivative of GPIC, strain CC13, was derived and evaluated in vitro and in vivo. Transcriptional profiling of CC13 revealed only partial conservation of previously identified plasmid-responsive chromosomal loci (PRCL) in *C. caviae*. However, 2-deoxyglucose (2DG) treatment of GPIC and CC13 resulted in reduced transcription of all identified PRCL, including *glgA*, indicating the presence of a plasmid-independent glucose response in this species. In contrast to plasmid-cured *C. muridarum and C. trachomatis*, plasmid-cured *C. caviae* strain CC13 signaled via TLR2 in vitro and elicited cytokine production in vivo similar to wild-type *C. caviae*. Furthermore, inflammatory pathology induced by infection of guinea pigs with CC13 was similar to that induced by GPIC, although we observed more rapid resolution of CC13 infection in estrogen-treated guinea pigs. These data indicate that either the plasmid is not involved in expression or regulation of virulence in *C. caviae* or that redundant effectors prevent these phenotypic changes from being observed in *C. caviae* plasmid-cured strains.

## Introduction

Chlamydiaceae are gram-negative obligate intracellular pathogens that infect ocular, genital and respiratory tissues in both humans and animals. The genomes of chlamydial species are highly conserved, which likely reflects the specific requirements of intracellular pathogens for survival and the limited opportunity for genetic exchange with other bacterial pathogens within this intracellular niche (reviewed by Stephens et al. [1]). This high degree of genetic-relatedness extends to carriage of a 7.5 kb “cryptic” plasmid by *Chlamydia trachomatis* (human), *C. muridarum* (mice), *C. psittaci (birds), C. felis* (cats), strains of *C. pneumoniae* that do not infect humans and *C. caviae* (guinea pigs*).* Plasmid-deficient *C. trachomatis* isolates are extremely rare, leading to speculation on the importance of the plasmid for chlamydial pathogenesis [2].

We developed a protocol for the derivation of plasmid-deficient chlamydiae and demonstrated its efficacy using *C. muridarum* [3]. The plasmid-cured derivative of *C. muridarum* Nigg, strain CM972, displays distinctive phenotypic changes in vitro and in vivo when compared with its parent. CM972 is less infectious in vitro [3] and this is associated with significantly reduced chlamydial load in the oviducts of intravaginally inoculated mice [4], [5]. In addition, CM972 lacks the ability to accumulate glycogen within the inclusion, a property shared by naturally occurring plasmid-deficient isolates [3], [6]. CM972 also did not cause oviduct pathology or signal via Toll-like receptor 2 (TLR2) [4], which is important for the development of oviduct pathology in this model [7]. Importantly, primary infection with CM972 prevented the development of pathology upon secondary challenge with wild-type Nigg. Recently, we demonstrated that these phenotypic changes are conserved in plasmid-cured *C. trachomatis* and identified a number of PRCL that, in addition to plasmid-encoded gene products, are candidate effectors of these virulence properties [8].

*C. caviae* is a natural pathogen of guinea pigs that causes inclusion conjunctivitis and respiratory infection in newborns. The guinea pig has been used to model sexual transmission of chlamydial infection from males to females [9] and to study genital tract infection and disease pathology in females [10], [11], [12]. Nevertheless, *C. caviae* is more distant genetically from *C. trachomatis* and *C. muridarum* and differs phenotypically from them in several respects including intrinsic resistance to sulphonamides [13], non-fusing inclusions [14] and an inability to accumulate glycogen [15]. We sought to examine the role of the conserved cryptic plasmid more broadly within the Chlamydiacae by examining the in vitro and in vivo consequences of curing the plasmid from *C. caviae*. We hypothesized that plasmid-deficient *C. caviae* would not activate TLR2 and would fail to cause oviduct disease in the guinea pig model. However, in contrast to plasmid-cured *C. muridarum and C. trachomatis*, plasmid-cured *C. caviae* strain CC13 signaled via TLR2 in vitro and elicited cytokine production in vivo similar to wild-type *C. caviae*. Furthermore, pathology induced by guinea pig genital tract infection with this strain was not reduced although we observed more rapid resolution of infection with CC13 in estrogen-treated guinea pigs. These data indicate that the association of the chlamydial plasmid with virulence is not universally conserved among chlamydial species.

## Methods

### Animals

Female 20-week-old outbred Hartley strain (Hilltop Labs, Scottdale, PA) guinea pigs were used for experiments. Guinea pigs were given food and water ad libitum in an environmentally controlled room with a cycle of 12 h of light and 12 h of darkness. All animals were determined to be seronegative for anti- *C. caviae* antibodies prior to infection. All animal experiments were pre-approved by the Institutional Animal Care and Use Committee of the University of Pittsburgh Medical Center under protocol # 0807981.

### Strains, cell lines and culture conditions

*C. caviae,* guinea pig inclusion conjunctivitis (GPIC) strain, was provided by Dr. Roger Rank and plaque-purified before use. Chlamydiae were cultured in L929 fibroblasts. Cells were infected at an approximate MOI of 0.5–1 before being centrifuged for 1 hour at 37°C. The cell culture medium was then removed and replaced with 1 X Dulbecco's modified Eagle's medium (DMEM) supplemented with 10% heat-inactivated FBS, gentamicin (20 µg ml^−1^) and 0.1 µg ml^−1^ cycloheximide. Infected cells were harvested into sucrose phosphate glutamate buffer at 40 hours post infection, sonicated, and maintained at −80°C. Bacteria were subsequently titrated by either the plaque assay [3] or as inclusion forming units (IFU) [16] using a genus-specific fluorescently tagged anti-chlamydial LPS monoclonal antibody (Biorad, Hercules, CA). Evaluation of plaquing efficiency under different culture conditions (e.g. with or without centrifugation) was achieved by the titration of individual strains by plaque-forming assay, with indicated modifications, in parallel with titration to estimate IFU. The efficiency of plaquing (EOP) was calculated as PFU ml^−1^ /IFU ml^−1^.

### Quantitative PCR

Total RNA was isolated from chlamydia-infected L929 cells 24 hours post-infection (MOI∼1) using an RNeasy RNA isolation kit (Qiagen, Valencia, CA). For quantitative PCR analysis, the RNA (2 µg) was pretreated with DNase I (Ambion) and cDNA prepared using an iScript cDNA synthesis kit (BioRad, Hercules, CA) according to the manufacturer's instructions. Primer pairs for amplification of 16S rRNA and the genes identified in this study (Table 1) were selected using Beacon Designer 2.13 (Premier Biosoft International, Palo Alto, CA). Primers were purchased from Integrated DNA Technology (Coralville, IA). Real time PCR reactions were carried out using iQ Sybergreen supermix (BioRad) in a BioRad iCycler using a two step reaction, 95°C for 10 seconds, 55°C for 1 minute for a total of 40 cycles. Melt curve analysis showed that the accumulation of SYBR green-bound DNA was gene specific and not caused by formation of primer dimers. Transcripts from the 16S rRNA gene of *C. caviae* served as an endogenous reference and data were analyzed by the 2^−ΔΔCT^ method [17] using BioRad proprietary software. Each sample was assayed in triplicate and each experiment was performed at least twice. Statistical significance was determined by using Student's t test. A *p* value of <0.05 was considered significant.

**Table 1:** PCR primer sets used in this study.

Strain	Locus^a^	Sense Primer	Anti-sense Primer
*C. caviae*	16S rRNA	GCTCCTGGAGTTTACAGTGAA	AAGAACGACGTTGGCGATAA
GPIC	CCA_00078 (*mip*)	TCCATTGCTTCCTGTGATTC	TTGCGTAACTGTCTTGCG
	CCA_00259	GAGGATAGGAAGGCTTGGAAC	CACAAACATGCAAGCTCTATCTTC
	CCA_00416	TTAGTGCGTGCTGGAGGAAG	ATTGGCGAGGCTACAGAACC
	CCA_00417	CTGTTGACCCCATAGAACCAC	GAGCCTAGAAACGACTCAGATG
	CCA_00453	GTTCTCAATGTCCGCAATACC	TCAGCAAACTATACACATACTTCC
	CCA_00523-525	GGGTTGGGATAATGGTTGG	AGTTGCTCTGATTAAGTATTGC
	CCA_00821 (*glgA)*	AAG ATGATACATTACGCTTCA GCG	TTAGAAGCCCCATATGCCAATCA
	CCA_00924	AACGGCGATCTCCTTATC	AAACCATAATTCCCAATCTCC
			
pCpGP1	pGP1-D	CAGGTCTTGCAGCGACAACA*	ACGTTCACCGTTCACGCTTA*
	pGP1-D	AGAAGCGCTAATAGCCCTTG	TAGACCTTCCCAGCCTTCAC
	pGP2-D	AACAGACGTTCACCGTTCAC	GCTGTTCTTCCCATCATCAC
	pGP3-D	GCGAAAGTTCCAGCTAGTTG	ATGAGGTTGAGCGGGTTATG
	pGP4-D	TGGTTGGCCACTTTATCTCC	GCATGCCTGCTTAACAACTC
	pGP4-D-pGP6-D	TAGGTTTGTTCCCCGACTCT*	GACTTGATCTGCCCTGTT*
	GP4/6 intrageneic	AACAGGGCAGATCAAGTC	GTTTGAGAAGCCCTCAAG
	pGP6-D	CATTCCTTCGTCGGTTCTTG	CCTAGACTTGGTGGAGTATC
	pGP7-D	TGTTGAGAAAGATTTCCCAGAACTAGA	TTGTTCGACTATAGGAGAGTCGGG
	pGP8-D	AACAACCTCCACACCAACAG	TAACTAACAGCGCAAGGAGC

a Chromosomal loci are identified by their annotated designations, *C. caviae* GPIC GenBank accession AE015925. Plasmid ORFs of pGPGP1 are indicated according to GenBank accession AE015926 [45].

*Plasmid primers used to detect presence of pCpGP1 by PCR or DNA hybridization only.

### In vitro analysis of TLR signaling

The following cell lines were examined: HEK293 cells stably expressing either TLR2 or TLR4/MD2 [18]. X-ray-irradiated preparations of chlamydiae were also assayed using HEK-TLR2 cells transfected with an NF- κB reporter plasmid as previously described [8]. In addition, in vitro infection was performed in murine bone marrow-derived dendritic cells (BMDDCs) cultured following the procedure of Inaba et al. [19]. The TLR2 agonist, Pam_3_Cys-Ser-(Lys)_4_ (Axxora, San Diego, CA), the TLR4 agonist LPS (Sigma, St. Louis, MO), and human rTNF-α (R&D Systems, Minneapolis, MN) were used as positive control stimulants. Cells were plated in 24-well tissue culture dishes at a density of ∼10^5^ cells/well. Infections were conducted by overlaying cells with a multiplicity of infection of 1 or 3. Cells were incubated for 24 hrs at 37°C, 5% CO_2_. Supernatants from the HEK cells were harvested and assayed for IL-8 (DuoSet, R&D Systems). The BMDDC supernatants were assayed for IL-1α, IL-1β, IL-2, IL-4, IL-5, IL-6, IL-9, IL-10, IL-12p70, IL-13, IL-17, G-CSF, GM-CSF, KC, MIP-1α, TNFα, IFNγ, and MCP-1 via Multiplex bead assay (Millipore Billerica, MA). IL-2, IL-4, IL-5, IFNγ, IL-9, IL-12p70, IL-13, MCP-1 and IL-17 were not detected above media (data not shown). BMDDC data points represent the mean levels of two independent experiments ± SD.

### Infection

Guinea pigs were inoculated intravaginally with 10^6^ IFU of either GPIC or CC13 suspended in 30 µl SPG intravaginally. Estradiol administration has been demonstrated to potentiate infection and pathology in the guinea pig model [20] so selected experimental groups were dosed with 1 mg sesame oil-emulsified β-estradiol-3-benzoate (10 mg/ml) (Sigma) daily where indicated, beginning 7 days before infection and continuing until sacrifice. Three separate experiments were conducted with groups of estrogen-treated animals sacrificed on days 9 or 30 and groups of untreated animals sacrificed on day 30. The kinetics of lower genital tract infection was monitored via culture of cervical swabs on L929 cells.

### In vivo cytokines

Guinea pig genital tract secretions were collected via vaginal sponges as described previously [21]. Sponges were stored at −70°C until they were eluted individually in 0.5 ml of Eagle minimal essential medium and assayed via ELISA for IL-8 (Human IL-8 DuoSet, R&D Systems).

### Serum antibody

IgG1, and IgG2a, and IgA antibody to *C. caviae* in serum was measured by an ELISA as described previously [22] using gradient purified GPIC elementary bodies as antigen and goat, anti-guinea pig-Ig antibodies obtained from AbdSerotech (Oxford, UK).

### Histopathology

Guinea pigs were sacrificed at day 9 or day 30 post infection, and the entire genital tract was removed en bloc, fixed in 10% buffered formalin, and embedded in paraffin. Longitudinal sections (4 µm) were stained with hematoxylin and eosin and evaluated by a pathologist blinded to the experimental design. Each anatomic site (exocervix, endocervix, uterine horn, and oviduct) was independently assessed for the presence of acute inflammation (neutrophils), chronic inflammation (lymphocytes/monocytes), plasma cells, and erosion of the mucosa. Right and left uterine horns and right and left oviducts were evaluated individually. A four-tiered semiquantitative scoring system was used to quantify the inflammation as previously described [5]. Oviduct dilatation scores also include a subjective assessment of flattening of the oviduct epithelial plicae and destruction of the oviduct mucosa.

### Statistics

Statistical comparisons between GPIC and CC13-infected guinea pigs for levels of infection and cytokine production over the course of infection were made using a two-factor (days and strain) RM ANOVA. A post-hoc Tukey test was used as a multiple comparison procedure. Kaplan-Meier survival analysis was used to compare the durations of infection over time. The Fisher-exact test was used for determination of significant differences in frequency of pathological characteristics between groups. One-way ANOVA on ranks was used to determine significant differences in the pathological data between groups. Statistical tests were performed using SigmaStat software with p<0.05 considered significant.

## Results

### Plasmid-deficient *C.caviae* CC13 displays normal infectivity

**Figure 1:**
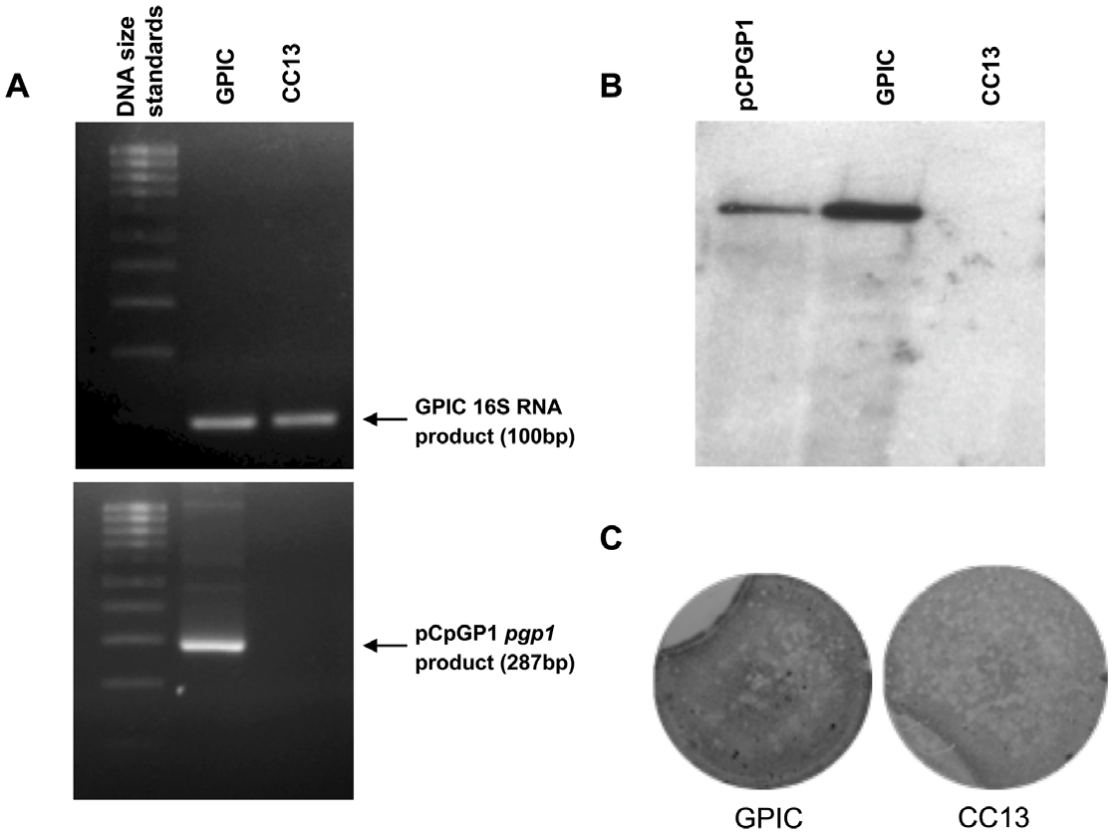
*C. caviae* CC13 is plasmid-deficient but displays no alteration in plaque size or efficiency. (A) Equal amounts of template were added to PCR reactions with primers directed against the pgp1 coding sequence expressed by pCpGP1 or the *C. caviae* genome and amplified products were analyzed on 2% agarose gels. (B) Genomic DNA was isolated from GPIC or CC13, digested with *Sph*I and separated on a 1% agarose gel. The DNA was transferred to a nylon membrane and probed for the presence of pCpGP1 DNA using a ∼1.6 Kb probe directed against a region spanning pGP4-6. Plasmid pCpGP1 was isolated from GPIC and digested with *Sph*1 to yield a single, linear fragment of ∼7.9 kb which was used as a positive control for hybridization. (C) Plaques formed by CC13 do not differ from those formed by GPIC. *C. caviae*-infected L929 monolayers were incubated for 5 days at 37 C, 5% CO2, then the media overlay was removed and the plaques visualized using crystal violet.

L929 cells infected with *C. caviae* GPIC were treated with novobiocin (62.5 µg/ml) and plated in a plaque assay as previously described for curing of the plasmid from *C. muridarum* [3]. Individual plaques were selected at random and each isolate was plaque-purified twice more in order to obtain clones. A total of 28 clones were screened by PCR using primers directed against *pgp1,* a gene encoded by the resident plasmid. Three plasmid-deficient clones were identified and one was selected for further analysis and designated strain CC13. Amplification and sequencing of a portion of the 16S rRNA gene from CC13 (Fig. 1A, Table 1) confirmed its genetic lineage, and Southern hybridization using a 1.58 Kb probe that spans the open reading frames encoding pGP4-6 of pCpGP1 confirmed the absence of the plasmid from this strain (Fig. 1B). PCR amplicons representing each of the plasmid-encoded open reading frames were detected in *C. caviae* GPIC only (Fig. S1).

*C. caviae* GPIC plaques were visible after 4–5 days incubation, and unlike plasmid-cured *C. muridarum* CM972 [23] or *C. trachomatis* CTD153 [8], no reduction in plaque size was observed for CC13 (Fig. 1 C). Furthermore, plaquing efficiency (EOP) in the absence of centrifugation was similar for GPIC (EOP = 2.77x10^−2^±1.94x10^−2^) and CC13 (EOP = 2.59x10^−2^±2.45x10^−2^), which contrasted with the reduced efficiency we previously observed for plasmid-cured *C. muridarum* [23] and *C. trachomatis* [8].

### Plasmid-responsive transcription by chromosomal loci is partially conserved in *C. caviae*

**Figure 2:**
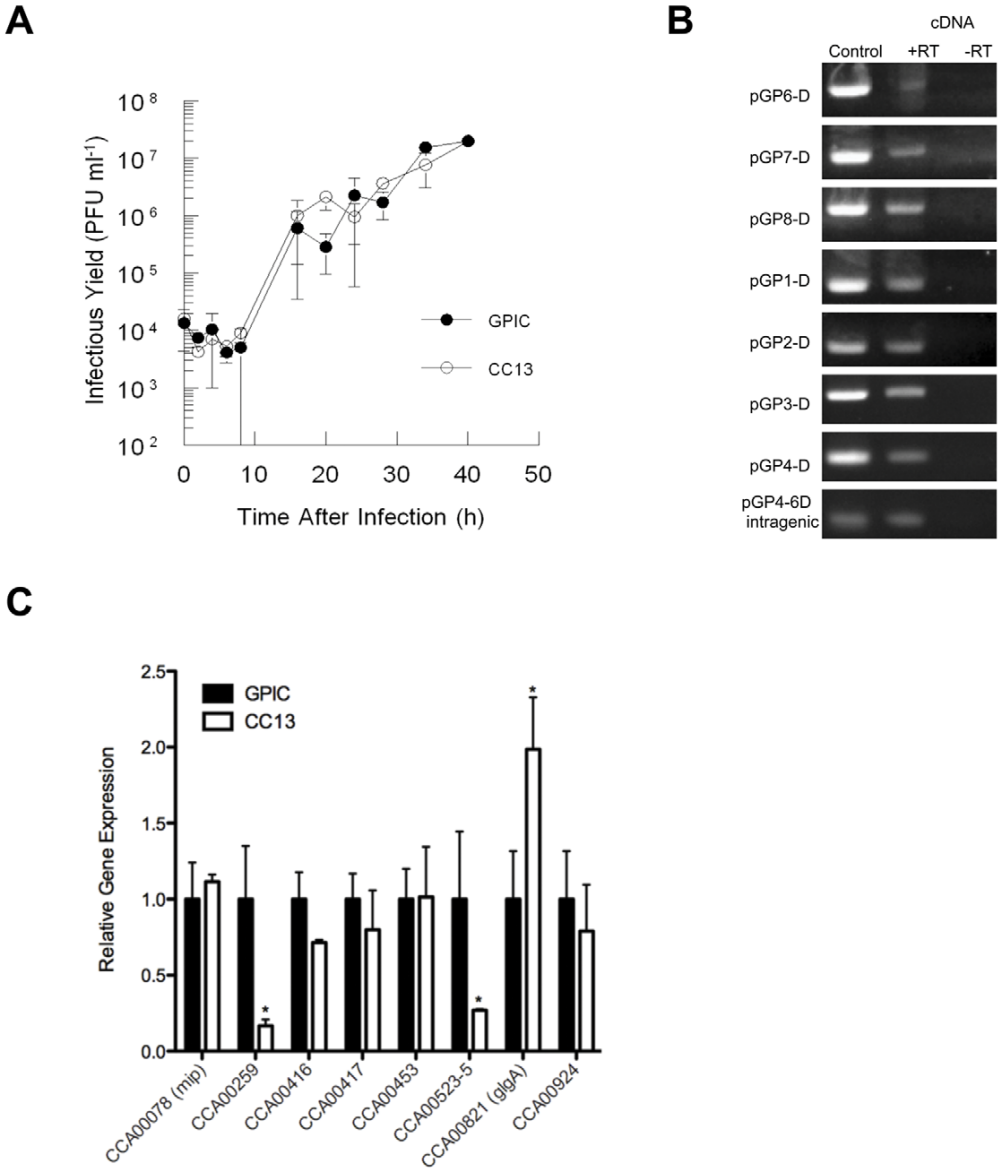
Transcriptional profiling of CC13 via microarray screening and quantitative RT-PCR reveals partial conservation of PRCL but a plasmid-independent glucose response in *C. caviae.* (A) CC13 replicates normally during synchronous infection of L929 cells. GPIC and CC13 were inoculated at an MOI ∼1 into L929 cells growing in 24 well dishes, then samples were harvested at intervals and titrated to determine IFU/well. (B) pCpGP1 transcripts are detected 24 hours after infection. Total RNA isolated from L929 cells 24 hours after infection with GPIC was treated with reverse transcriptase to generate cDNA and assayed via RT-PCR. Purified genomic GPIC DNA was used as positive control for the reactions while RNA processed for cDNA without reverse transcriptase was used as the negative control. (C) Quantitative RT-PCR confirms partial conservation of candidate PRCL in *C. caviae*. Total RNA was isolated from infected cells 24 hours after infection and PRCL transcripts were measured by quantitative RT-PCR. CCA00078 (*mip*) was included as a non-plasmid responsive control because we did not detect a difference in its transcription by microarray screen.

Comparison of the transcriptional profiles of plasmid-cured strains of *C. muridarum* and *C. trachomatis* revealed a conserved group of plasmid-responsive chromosomal loci (PRCL) whose transcription was altered in the absence of the plasmid [8]. To determine if similar transcriptional changes have occurred in the plasmid cured CC13 we first confirmed that the absence of the plasmid does not impact the growth rate of CC13 when compared to GPIC (Fig. 2A). Furthermore, RT-PCR confirmed that transcripts of all plasmid-encoded ORFs could be detected by 24 hours after infection (Fig. 2B). Interestingly, both strains resembled *C. muridarum* Nigg [4] in developmental profile because new GPIC and CC13 EBs were detected in tissue culture by 16 hours post infection. Real time RT-PCR analysis of PRCL homologs expressed by *C. caviae* revealed significantly decreased transcription of several, but not all, of these genes at 24 hours post infection, including the putative operon encoding CCA00523-525 and CCA00259 (Fig. 2C). Transcription of CCA00416 and CCA00417, that encode orthologs of Pls1 and Pls2 [24] respectively appeared only mildly reduced 24 hours after infection (Fig. 2C). In contrast, transcription of CCA00453, encoding a phospholipase D enzyme [25] and CCA00924 was not significantly altered in CC13 (Fig. 2C) although all four of these genes were plasmid-responsive in *C. muridarum* and *C. trachomatis* [8]. Transcription of *glgA*, the gene encoding glycogen synthase, is plasmid-dependent in *C. trachomatis* [26] but not in *C. muridarum* [8]. Transcription of *glgA* by CC13 was mildly increased whether detected by microarray (data not shown) or by quantitative PCR (Fig. 2C). A microarray screen comparing the transcriptional profile of GPIC with CC13, 30 hours after infection confirmed these findings (Fig. S2) and failed to detect additional plasmid-responsive loci with orthologs in *C. trachomatis* and *C. muridarum*.

**Figure 3:**
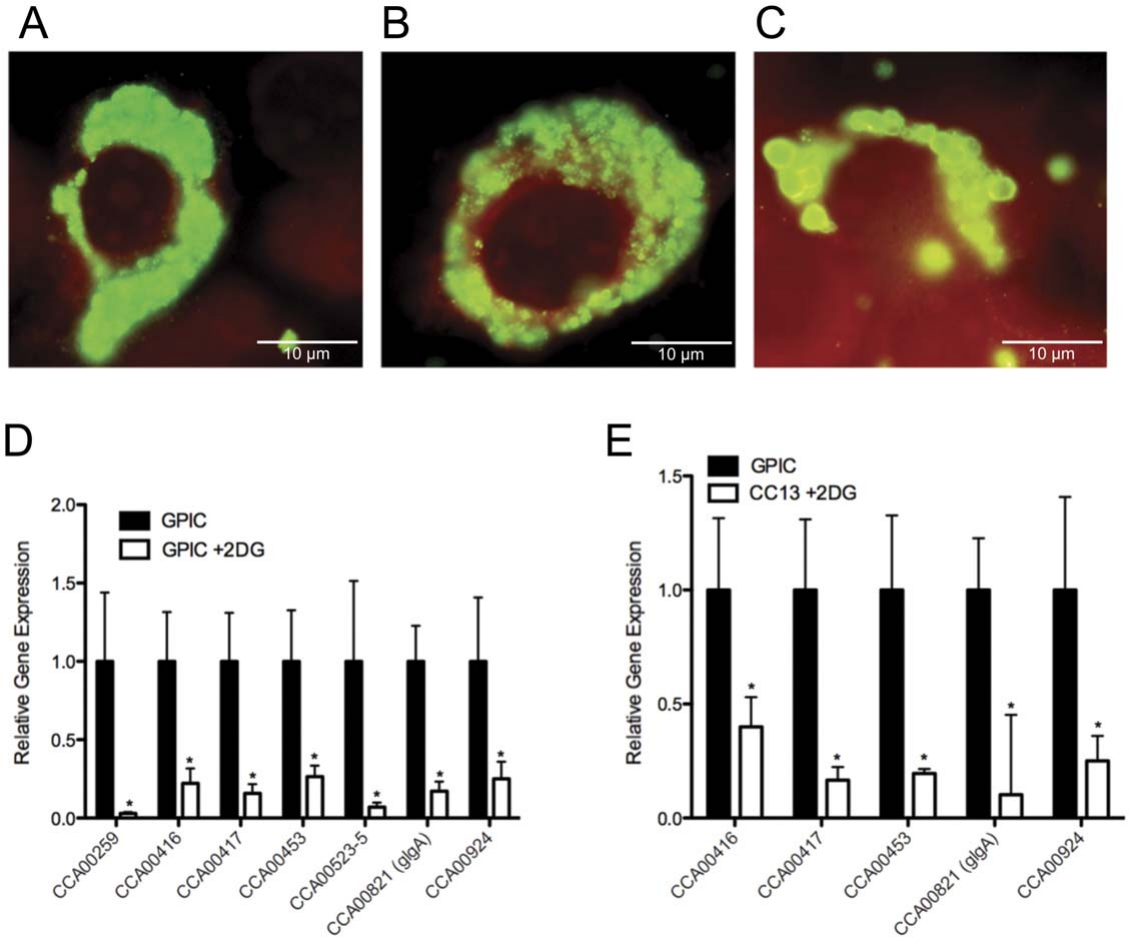
Effect of 2DG treatment on inclusion formation and transcription by *C. caviae*. (A) GPIC inclusions in L929 cells 40 hours post infection (A–C). Inclusions formed by GPIC in cells treated with 10 mM 2DG are smaller (B) and may contain aberrant forms (C). Cells were fixed with methanol then stained with an anti-LPS monoclonal antibody and anti-mouse Alexa488 (secondary) to detect chlamydiae and cytoplasm was counterstained with Evans Blue. All inclusions were imaged at 400x magnification. (D) GPIC differentially regulates candidate PRCL transcription in response to 2DG treatment. Total RNA was isolated 24 hours after infection from infected cells treated with 10 mM 2DG and transcripts were quantified as for Fig. 2D. (E) Reduced PRCL transcription is plasmid-independent in *C. caviae*. L929 cells infected with CC13 were treated with 2DG as for ‘C’ and transcription of plasmid-insensitive loci 24 hours after infection was examined as before. The transcriptional differences are presented as fold change in expression for each gene in a single representative experiment although each experiment was performed independently at least twice and samples were assayed in triplicate. *p* values for transcripts indicated with * were <0.05.

Transcription of *C. trachomatis* PRCL was coordinately reduced in response to glucose limitation [8]. We examined the impact of 2DG treatment on the development of GPIC inclusions. L929 cells infected with GPIC were cultured with medium containing the glucose-6-phosphate inhibitor 2-deoxyglucose (2DG). Inclusions formed by GPIC in the presence of 10mM 2DG appeared smaller, with fewer EBs (Fig. 3B) when compared with those formed in untreated cells (Fig 3A). Occasionally inclusions containing larger aberrant forms were observed (Fig. 3C) that were not noted in the untreated cells. Overall, the number of inclusions was reduced and the infectious yield dropped ∼100 fold after treatment (data not shown). Treatment of GPIC-infected cells with 2DG also resulted in significantly reduced transcription of *glgA* (∼6 fold reduction) and other PRCL homologs (4.5–34.4 fold reduction) (Fig. 3D). Transcription of CCA00416, CCA00417, CCA00453 and CCA00924 was also significantly reduced in CC13 in response to treatment with 2DG (Fig. 3E) indicating that glucose-responsiveness by *C. caviae* was not dependent on the presence of the plasmid. These observations suggested a reduced role for pCpGP1 in regulation of chromosomal loci in *C. caviae* and indicated that plasmid-deficient *C. caviae* may not express phenotypes described for plasmid-deficient *C. muridarum* and *C. trachomatis*.

### *C. caviae* CC13 activates TLR2 signaling in vitro

**Figure 4:**
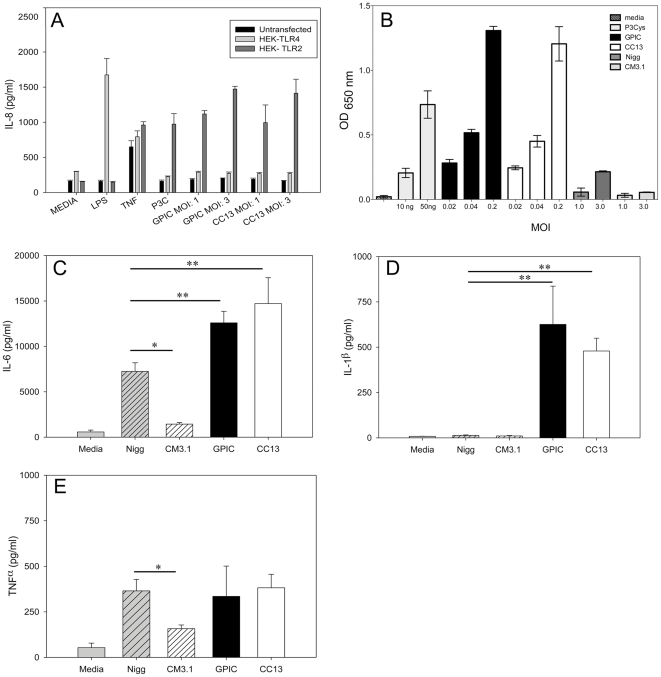
CC13 signals via TLR2 and induces cytokine production at levels similar to wild-type GPIC. (A) IL-8 was measured in the supernatants of HEK 293 cells transfected with control plasmid, TLR2 or TLR4 and infected with GPIC or CC13 for 24 hrs. At both an MOI of 1 and 3, cytokine production did not differ between HEK-TLR2 cells infected with GPIC or CC13. Neither strain induced IL-8 levels above media for cells transfected with control plasmid or TLR4. The pattern of IL-8 production after stimulation with LPS (TLR4 agonist), TNF (NF-κB agonist), or Pam_3_Cys (TLR2 agonist) indicated that cytokine production was specific for the transfected TLR. (B) GPIC and CC13 express a potent, plasmid-independent TLR2 stimulating activity. X-ray inactivated chlamydial suspensions at various MOIs were incubated with HEK-TLR2 cells transfected with an NF-κB reporter plasmid. After 24 h of incubation NF-κB-induced secreted alkaline phosphatase activity was assayed using QUANTI-Blue. Bars represent the mean ± SE for three independent experiments. (C) Murine BMDDCs infected with GPIC or CC13 for 24 h did not differentially produce IL-6, (D) IL-1β, or (E) TNF-α. Bars represent the mean ± SD for duplicate wells. The data presented are from a single representative experiment that was performed at least twice.

Plasmid-deficient *C. muridarum* and *C. trachomatis* do not signal via TLR2, and induce production of significantly lower levels of proinflammatory cytokines than wild-type strains [4]. We infected HEK 293 cells stably expressing TLR2 (HEK-TLR2) or TLR4/MD2 (HEK-TLR4) with *C. caviae* GPIC or CC13. HEK 293 cells endogenously express TLR1, TLR6 and MyD88, but not TLR2 or TLR4 [18], thus, specific signaling can be determined by measuring cytokine production in cells stably transfected with TLR2 [23] because TLR-ligand interactions are highly conserved in nature [18]. Analysis of supernatants after 24 hours incubation revealed similar levels of IL-8 production by HEK-TLR2 cells in response to infection with GPIC or CC13 (Fig. 4A). IL-8 production above media was not detected in the supernatant of HEK-TLR4 cells infected with either strain, consistent with the weak immunostimulatory capabilities of chlamydial LPS [27]. These data indicate that despite absence of the plasmid, CC13 was able to induce TLR2-dependent cytokine production at levels equivalent to GPIC. Interestingly, while we observed no difference between TLR2 activation by CC13 or GPIC when we assayed X-ray inactivated preparations of the bacteria using HEK-TLR2 cells transfected with an NF- κB reporter plasmid (Fig. 4B), we noted that the amount of *C. caviae* required to activate TLR2 in these cells was considerably lower (∼100 fold reduced) when compared with *C. muridarum* Nigg or its plasmid-cured derivative CM3.1, suggesting that the TLR2 ligands of this chlamydial species is extremely potent.

### *C. caviae* is highly pro-inflammatory when compared with *C. muridarum* in murine BMDDCs

*C. muridarum* plasmid-deficient strains induce significantly less cytokine production by dendritic cells in vitro than their wild-type parent [4]. We incubated murine bone marrow derived dendritic cells (BMDDCs) with CC13, GPIC, *C. muridarum* Nigg or CM3.1 to determine if dendritic cell cytokine release was altered by the loss of the plasmid from *C. caviae* and to compare cytokine release during infection with *C. caviae* or *C. muridarum*. After 24 hours, BMDDCs stimulated with GPIC and CC13 secreted IL-1α, IL-1β, IL-6, IL-10, G-CSF, TNF-α, GM-CSF, MIP-1α, and KC. Strikingly, IL-6 (Fig. 4C), IL-1β (Fig. 4D), IL-1α, IL-10, MIP-1α, and KC (data not shown) were secreted at significantly higher levels in response to infection with *C. caviae* compared to *C. muridarum*, whereas G-CSF (data not shown), GM-CSF (data not shown), and TNF-α (Fig. 4E) levels were similar between the strains. In all instances, we observed no difference in cytokine response to infection with CC13 compared with GPIC. These data indicate that CC13 stimulated dendritic cell cytokine production to levels equivalent to GPIC and contrasted with *C. muridarum* where wild-type Nigg induced significantly greater cytokine release than plasmid-deficient CM3.1.

### *C. caviae* CC13 is not attenuated in the guinea pig genital tract infection model

**Figure 5:**
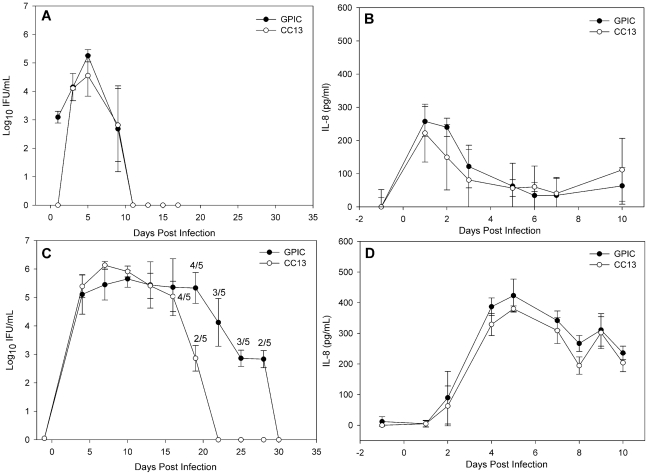
Chlamydial burden and levels of IL-8 do not differ between GPIC and CC13 infected guinea pigs during genital tract infection. Groups of 5 untreated (A, B) or estradiol-treated (C, D) female guinea pigs were intravaginally infected with either GPIC or CC13 and sacrificed on day 30. (A) Monitoring of endocervical swabs during infection of untreated guinea pigs revealed that infectious bacteria were not detectable after day 9 for either strain. (B) Vaginal sponges collected through day 10 revealed similar levels of IL-8 in GPIC and CC13-infected guinea pigs. (C) The course of GPIC infection in estradiol-treated guinea pigs was prolonged compared to CC13. (D) IL-8 levels monitored during the first 10 days of infection were not different in estradiol-treated guinea pigs infected with GPIC and CC13. Bars represent the mean ± SD of five guinea pigs per group and each experiment was performed once.

In order to determine the possible impact of plasmid-deficiency on the virulence of *C. caviae* in the genital tract infection model, groups of guinea pigs (N = 5) were inoculated intravaginally with either GPIC or CC13 and sacrificed on day 30. The course and intensity of infection was monitored via endocervical swabs. No difference in the magnitude or duration of infection was noted between the strains (Fig. 5A). In addition, IL-8 levels measured in genital secretions obtained during the first 10 days of infection were similar in the two groups (Fig. 5B). Histologic examination of oviduct tissue recovered from these guinea pigs failed to detect any pathology regardless of infecting strain.

Treatment with estrogen maintains the reproductive tract epithelium and results in dramatically increased bacterial burden [28], which might facilitate detection of subtle differences in the course of infection and outcome with respect to upper reproductive tract pathology between CC13 and its plasmid-containing parent GPIC. Groups of estradiol-treated female guinea pigs (N = 5) were intravaginally inoculated with GPIC or CC13 and sacrificed on day 9 or day 30 post-infection. Culture of endocervical swabs again revealed no significant difference (two-way RM ANOVA, *p* = 0.898) in shedding of chlamydiae from the lower genital tract between GPIC and CC13 infected animals (Fig. 5C). However, GPIC infection was prolonged compared to CC13 (Kaplan-Meier, *p* = 0.02), with infection resolving by day 22 for CC13 and day 30 for GPIC (Fig. 5C). Swabs obtained from the upper uterine horns of guinea pigs sacrificed on day 9 were titrated via plaque assay and the bacterial burden did not differ significantly (*p* = 0.052, Student's t-test) between the strains (GPIC: 9.8×10^4^±4.1×10^4^; CC13: 4.3×10^3^±2.1×10^3^ PFU/ml). Thus, in estradiol-treated animals, the duration of genital tract infection was shortened and upper genital tract bacterial burden was slightly decreased during infection with CC13.****

However, we were unable to detect differences in the cytokine response to infection between estradiol-treated guinea pigs infected with GPIC or CC13. Genital tract secretions collected from CC13 and GPIC infected animals through day 10 contained similar levels of IL-8 (Fig. 5D). Examination of the antibody response to infection in the estradiol-treated animals was performed using serum collected at the time of sacrifice on day 30. Analysis of antibody titers revealed no difference in the mean ± SD log10 titers of IgG1 (GPIC, 3.94±0.38 vs. CC13, 3.51±0.35), IgG2a (GPIC, 3.89±0.34 vs. CC13 3.59±0.46) or IgA (GPIC, 2.81±0.3 vs. CC13, 2.81±0.42) between the strains. These data indicate that despite the mildly prolonged infection observed during infection with GPIC, the humoral immune response did not significantly differ from that of animals infected with CC13.

### Estradiol-treated guinea pigs exhibit similar inflammatory scores and pathology during infection with GPIC or CC13

**Figure 6:**
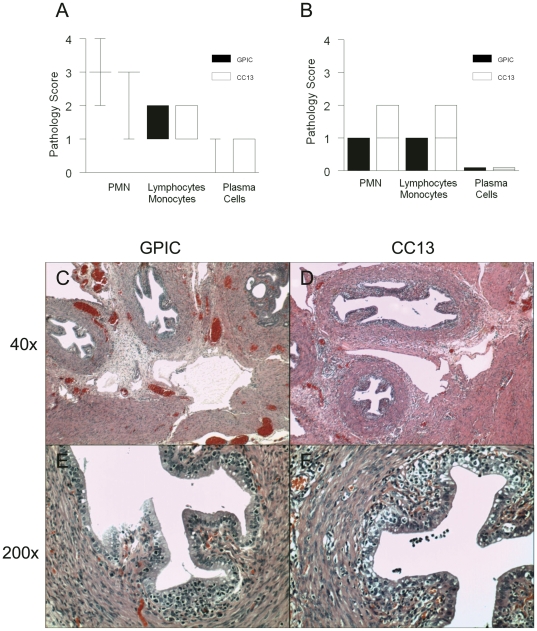
Cellular infiltrates are similar early during infection in estradiol-treated guinea pigs infected with GPIC or CC13. Groups of 5 estradiol-treated female guinea pigs infected with GPIC or CC13 were sacrificed on day 9. Pathology scores for inflammatory cells in the (A) uterine horns, and (B) oviducts were similar for GPIC- and CC13-infected animals. Boxes extend from the 25-75 percentiles and whiskers indicate the 5^th^–95^th^ percentiles. (C, E) Representative oviduct histologic sections at 40X and 200X magnification from a guinea pig infected with GPIC. (D, F) Representative oviduct histologic sections at 40X and 200X magnification from a guinea pig infected with CC13. Data are from one experiment with 5 guinea pigs per group.

**Figure 7:**
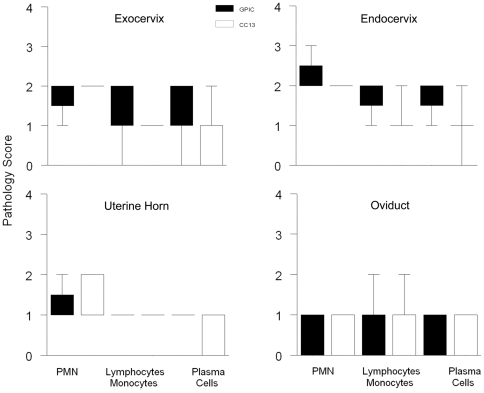
Cellular infiltrates are similar late during infection in estradiol-treated guinea pigs infected with GPIC or CC13. Groups of 5 estradiol-treated female guinea pigs were infected with GPIC or CC13 were sacrificed on day 30. Median pathology scores for inflammatory cells in the (A) exocervix, (B) endocervix (B), (C) uterine horns, and (D) oviducts were similar for GPIC- and CC13-infected animals. Boxes extend from the 25–75 percentiles and whiskers indicate the 5^th^–95^th^ percentiles. Data are from one experiment with 5 guinea pigs per group.

**Figure 8:**
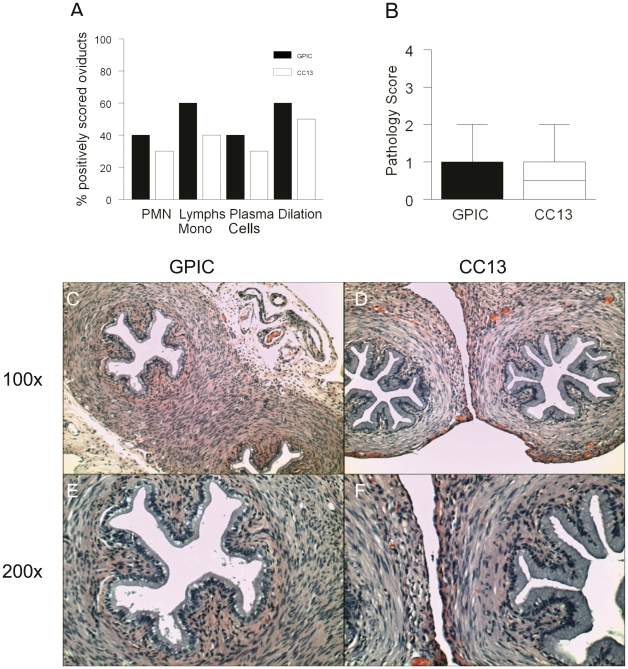
Oviduct pathology is similar following infection with GPIC or CC13. Groups of 5 estradiol-treated female guinea pigs were infected with GPIC or CC13 and sacrificed on day 30. (A) Bars represent the percentage of oviducts with a pathology score of ≥ 1 for degree of infiltration of polymorphonuclear neutrophils (PMNs), lymphocyes/monocytes, plasma cells or oviduct dilatation. (B) Median pathology scores for oviduct dilatation in GPIC- and CC13-infected guinea pigs. Boxes extend from the 25–75 percentiles and whiskers indicate the 5^th^–95^th^ percentiles. (C) Histologic sections of an oviduct from a representative estradiol-treated guinea pig on day 30 after infection with (C, E) GPIC or (D, F) CC13 shown at 100X and 200X, respectively. The oviduct architecture is intact with minimal inflammation and dilatation. Data are from the same experiment detailed in Fig. 6.

Genital tract tissues harvested from estradiol-treated guinea pigs infected with GPIC or CC13 were analyzed histologically. The groups sacrificed on day 9 post-infection were graded for cellular infiltrates because infection was still present. The groups sacrificed on day 30 were analyzed for cellular infiltrates and for pathology remaining after clearance of infection. On day 9, the levels of inflammation in the exocervix (data not shown), endocervix (data not shown), uterine horns (Fig. 6A), and oviducts (Fig.6B-F) did not differ between the strains. On day 30, histologic examination did not reveal any difference in the degree of inflammation in the exocervix (Fig. 7A), endocervix (Fig. 7B), uterine horns (Fig. 7C) or oviducts (Fig. 7D; Fig. 8 A–F) during infection with GPIC or CC13. In addition, there was no difference in the percentage of animals with oviduct dilatation (Fig. 8A) or degree of dilatation between the groups (Fig. 8B–F).

The overall degree of oviduct dilatation and oviduct epithelial cell damage resulting from genital tract infection was low. In contrast, we observed severe abdominal pathology in 3 of 5 GPIC-infected and 1 of 5 CC13-infected animals that were treated with estradiol, with fibrous adhesions noted between the genital tract and peritoneum, bowel, and bladder in these animals. These findings had been previously described in estrogen-treated, GPIC-infected guinea pigs by Rank et al [28] and are reminiscent of Fitz-Hugh-Curtis syndrome observed in a subset of Chlamydia-infected human females [29]. This syndrome results from migration of the bacterium into the abdomen and leads to inflammation and fibrous adhesions in infected women.

## Discussion

Our studies [4], [8] and others [26] have revealed an important role for the chlamydial plasmid in the expression of key virulence properties by both *C. muridarum* and *C. trachomatis*. However the role of the resident plasmid in other Chlamydiaceae has not been investigated. Strains of *C. pneumoniae* infecting humans generally lack the plasmid, but it is present in strains that infect a diverse range of other mammals including horses [30] and koalas [31]. Recent studies of *C. felis* clinical isolates indicate that plasmid carriage is highly conserved [32] in this species suggesting that the plasmid may be important for virulence, and although the plasmid appears conserved in *C. psittaci*, plasmid-deficient strains have been described [33]. In this study we investigated the role of the plasmid in *C. caviae*, a natural pathogen of the guinea pig, by curing GPIC of pCpGP1 to derive strain CC13 and by examining CC13′s ability to cause infection and genital tract disease.

Three plasmid-associated phenotypes have been identified in *C. muridarum* and are conserved in *C. trachomatis:* plasmid-deficient strains are unable to accumulate glycogen within the intracellular inclusion during the developmental cycle, display reduced infectivity in vitro [3], and in vivo [4] and do not stimulate TLR2 signaling during infection [4]. Whether the effectors of these phenotypes are encoded directly by the plasmid is unknown, but we have identified a conserved group of plasmid-responsive loci encoded on the chromosome that may also contribute to the expression or regulation of these traits [8]. Microarray screening using a custom GPIC array indicated that the transcriptional profile of CC13 very closely resembled that of its parent, but we nevertheless observed that several of the PRCL identified in plasmid-deficient *C. muridarum and C. trachomatis* were also differentially transcribed in CC13 including CCA00523-525 (orthologous to CT142-44 and TC_419-421), and CCA00259 (orthologous to CT382.1). However, other candidate PRCL such as the CT084 (TC_0357) ortholog CCA00453, and the CT702 (TC_075) ortholog CCA00924 did not differ transcriptionally from GPIC. Mild reduction in transcription of CCA00416-17 (orthologous to CT049-50 and [TC_319-320]) was detected that did not reach significance. Furthermore, transcription of *glgA* appeared slightly but significantly elevated (∼2 fold) in *C. caviae* CC13. The significance of this observation is unclear because glycogen accumulation within wild-type *C. caviae* inclusions is not observed and glycogen production by this chlamydial species has not been detected [34] but indicates that *glgA* transcription is not plasmid-dependent in *C. caviae,* more closely resembling what we have previously observed for *C. muridarum.*

Phenotypic analysis of CC13 in vitro revealed that loss of pCpGP1 did not impact plaque size or plaquing efficiency. GPIC, in common with *C. pneumoniae* and *C. psittaci*, does not accumulate glycogen intrainclusionally [15], so no change in iodine-staining phenotype was anticipated and was not observed (data not shown). Most significantly, CC13 retained the ability to activate TLR2 expressed on stably transfected HEK293 epithelial cells, an observation that contrasted with the plasmid-cured strains CM972 and CTD153 that are unable to stimulate TLR2-dependent signaling in vitro and in vivo [4], [8]. The overall conservation of chlamydial plasmid organization suggests that the plasmid may not encode a pathogenic TLR2 ligand directly and further, that the conservation of plasmid-responsiveness for CCA00523-25 and CCA00259 indicates that their expression is likely not required for TLR2 activation, reducing the likelihood that their orthologs encode candidate TLR2 ligands in *C. trachomatis* or *C. muridarum*. Alternatively, is also possible that GPIC encodes additional TLR2 ligands that are unaffected by the absence of the plasmid, preventing detection of differential TLR2 signaling effects as are observed in plasmid-cured *C. trachomatis* and *C. muridarum.* In support of this hypothesis, we observed that GPIC and CC13 activated TLR2 ∼100 fold more effectively than *C. muridarum* Nigg, reflecting the expression of a potent, plasmid-independent TLR2 ligand by *C. caviae*.

CC13 and GPIC both displayed a strongly pro-inflammatory profile in dendritic cells. Consistent with our observation that *C. caviae* expresses a potent, plasmid-independent TLR2 ligand we detected strong induction of both TNF-α and IL-6 by BMDDCs in response to incubation with GPIC and CC13. *C. caviae* strongly induced IL-1β production by BMDDCs while *C. muridarum* did not. Prestimulation with TLR ligands is required for IL-1β production during infection of macrophages with *C. muridarum* [35]. The high levels produced by BMDDCs infected with GPIC or CC13 suggests *C. caviae* is able to independently prime and induce release of IL-1β in these cells, unlike *C. muridarum*. Thus, it appears that in addition to a lack of plasmid-control for TLR2 activation, stimulatory pathways are activated by *C. caviae* differentially when compared to *C. muridarum.*

In light of our discovery that TLR2 signaling and infectivity were unimpaired in CC13 despite the absence of pCpGP1, it was not surprising that we were unable to demonstrate any significant attenuation in the guinea pig model of genital tract infection. Only with the potentiating effects of estradiol treatment were we able to detect a minor shortening in the course of infection and a slight reduction in upper genital tract bacterial load. Ultimately, these differences were not sufficient to drive differences in the development of oviduct immunopathology. Intra-abdominal adhesions were noted in animals in both groups and were likely the result of prolonged inflammation caused by the enhanced bacterial burden associated with administration of estradiol.

**Figure 9:**
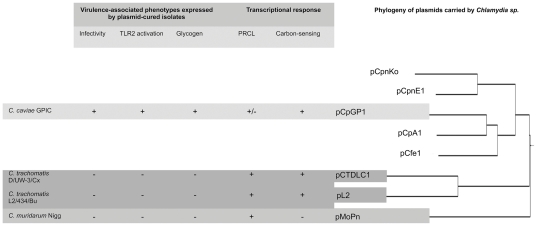
The impact of plasmid-curing on *Chlamydia sp*. gene expression, regulation and virulence. This diagram outlines the gene expression and regulation and virulence phenotypes of plasmid-cured *Chlamydia sp.*, and indicates the phylogenetic relationships of the plasmid carried by these species or strains and other genera. Data regarding phenotypic and transcriptional responses were obtained in the course of this or previous studies [3], [4], [5]. NT indicates where phenotype was not examined. The dendrogram was constructed via CLUSTAL [41] and Neighbor-joining analysis was performed using plasmid sequences from *C. trachomatis* D/UW-3/Cx (pCTDLC1) [42], L2/434/Bu (pL2) [43], *C. muridarum* (pMoPn) [44], *C. caviae* (pCpGP1) [45], *C. psittaci* pCpA1 (Lusher,M.E., Gregory,J., Storey,C.C. and Richmond,S.J., unpublished; Genebank: NC_002117, *C. felis* (pCfe1) [46] and the non-human pathogens *C. pneumoniae* LPCoLN [31] and N16 (pCpnE1) [47] to determine branching order. The outcome of this analysis did not differ from the more detailed analysis published by Mitchell et al. [31].

Comparisons of the effects of plasmid-curing on *C. trachomatis*, *C. muridarum* and *C. caviae* gene expression, regulation and virulence indicate significant differences that may be important for understanding the outcome of genital tract infection with these pathogens (Fig. 9). The overall similarity of both plasmid organization and sequence homology is high. Recent phylogenetic analysis of the plasmids expressed by 6 of the 9 chlamydial species indicates that they group distinctly, in a manner greatly resembling their genomes, with the plasmids obtained from *C. pneumoniae* strains most closely related to each other and to a lesser extent to those carried by *C. psittaci*, *C. felis* and *C. caviae*, and finally *C. muridarum* and *C. trachomatis* isolates [31]. With such genetic similarity, how can we account for the differences in phenotype that we have observed in plasmid-cured strains? If the plasmid encodes effectors of chlamydial virulence directly, why aren't these phenotypes conserved in all species that carry the plasmid? Carlson et al. [26] proposed that the plasmid encodes a transcriptional regulator, and we have identified a conserved sub-population of chromosomal loci that are under its control [8]. However, we observed only partial conservation of plasmid-dependence for these genes in *C. caviae* and if these are effectors of these phenotypes in *C. muridarum* and *C. trachomatis,* then this is a likely explanation of the failure of plasmid-curing to alter *C. caviae* virulence.

Interestingly, we observed significant reduction of all candidate PRCL transcription in GPIC in response to 2DG treatment, indicating that *C. caviae*, like *C. trachomatis*, alters gene expression in response to an environment in which glucose is limiting. Furthermore, this response persisted in the cured CC13 strain indicating that this process is plasmid independent. Consequently, *glgA* expression was plasmid-insensitive but glucose-limited by *C. caviae*, a novel transcriptional profile that contrasts with our observations for both *C. trachomatis* where *glgA* transcription is both plasmid and glucose sensitive and *C. muridarum* where *glgA* transcription is unaltered in response to plasmid loss or glucose limitation [8]. This may reflect a glucose-responsive regulatory pathway evolving within *Chlamydiaceae* to facilitate modulation of non-essential, plasmid-associated, virulence gene expression. This is a common theme in bacterial pathogens where expression of virulence loci may be tightly controlled in response to environmental signals such as temperature [36], nutrient limitation [37], carbon availability [38], or phosphate homeostasis [39]. In the context of such a model, it appears that *C. muridarum* has not co-evolved or has dispensed with the transcriptional controls exerted by this pathway with the result that transcription of PRCL remains constitutively active in glucose-limiting conditions [8]. If true, this may explain the high incidence of upper reproductive tract pathology observed in the mouse model [21] while human infection is predominantly sub-clinical and reproductive tract sequelae relatively uncommon [40]. Indeed, it may be worth noting that neither *C. caviae* nor *C. muridarum* are natural pathogens of the genital tract, infecting the eye and respiratory tract of their respective hosts, so coordination of virulence-associated gene expression in response to glucose availability might not be relevant for these sites. Our preliminary observations indicate that TLR2 signaling by *C. trachomatis* but not by *C. muridarum* is impaired when the chlamydiae have been cultured under glucose-restricted conditions [8], but until the pathogenic TLR2 ligand(s) have been identified, limited or altered expression of the TLR2 ligand(s) cannot be confirmed. If correct, then identification and characterization of the regulatory factor(s) encoded by the plasmid and those involved in the chlamydial carbon response will greatly advance our understanding of this important virulence process.

## Supporting Information

Figure S1 **Primers directed against the predicted open reading frames encoded on pCpGP amplify predicted fragments from** ***C. caviae*** **GPIC but not from CC13.** Primers pairs directed against each ORF are detailed in Table 1 and amplification conditions are described in Methods.
(DOCX)

Figure S2 **Scatter plot illustration of microarray comparison of the transcriptional profile of** ***C. caviae*** **GPIC and its plasmid-cured derivative CC13 30 hours after infection.**
(DOCX)
