# Supplementary material for: Plasmid-Cured Chlamydia caviae Activates TLR2-Dependent Signaling and Retains Virulence in the Guinea Pig Model of Genital Tract Infection
Source: PLoS One. 2012 Jan 24;7(1):e30747. doi: 10.1371/journal.pone.0030747 (PMC3265510; doi:10.1371/journal.pone.0030747)
Supplement: Figure S1 — Primers directed against the predicted open reading frames encoded on pCpGP amplify predicted fragments from C. caviae GPIC but not from CC13. Primers pairs directed against each ORF are detailed in Table 1 and amplification conditions are described in Methods. (DOCX) [file pone.0030747.s001.docx]

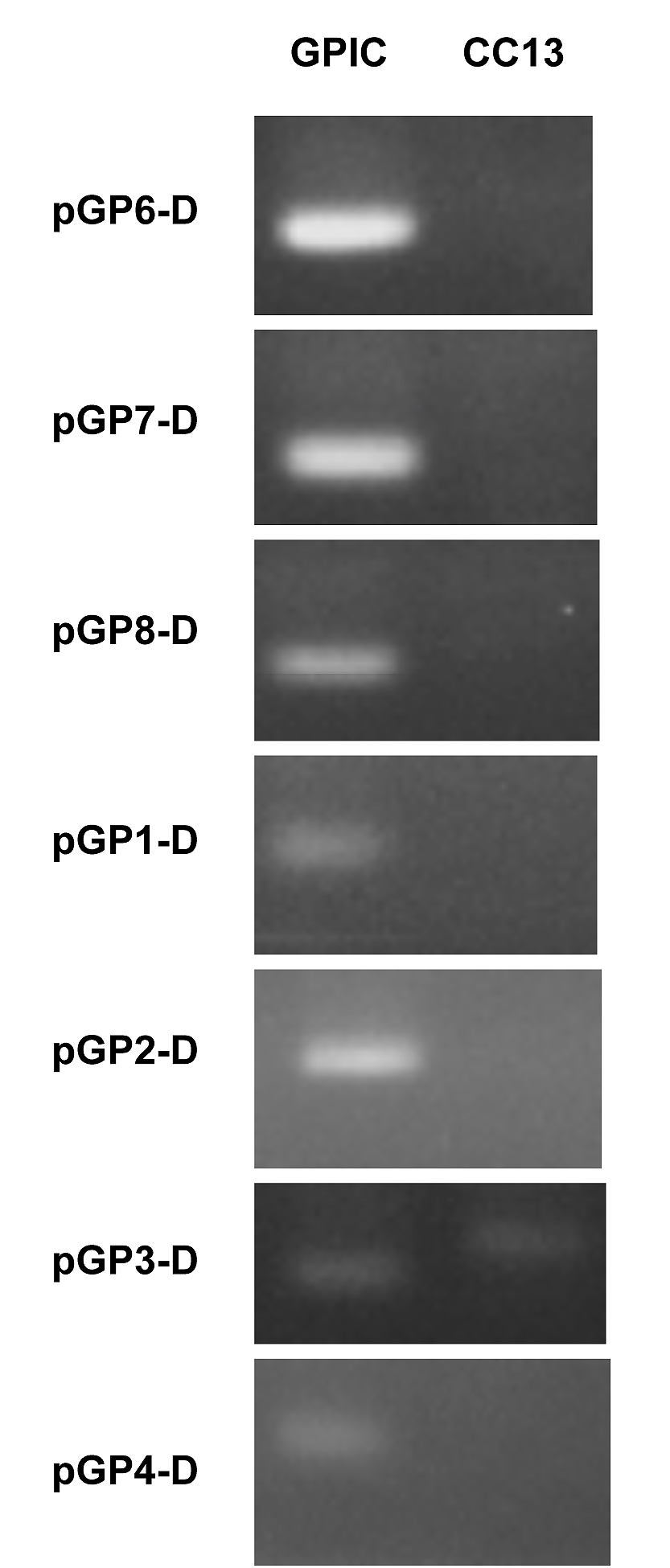
Supplemental Figure 1.

Primers directed against the predicted open reading frames encoded on pCpGP amplify predicted fragments from *C. caviae* GPIC but not from CC13. Primers are detailed in Table 1 and amplification conditions are described in Methods.
