# Supplementary material for: Plasmid-Cured Chlamydia caviae Activates TLR2-Dependent Signaling and Retains Virulence in the Guinea Pig Model of Genital Tract Infection
Source: PLoS One. 2012 Jan 24;7(1):e30747. doi: 10.1371/journal.pone.0030747 (PMC3265510; doi:10.1371/journal.pone.0030747)
Supplement: Figure S2 — Scatter plot illustration of microarray comparison of the transcriptional profile of C. caviae GPIC and its plasmid-cured derivative CC13 30 hours after infection. (DOCX) [file pone.0030747.s002.docx]

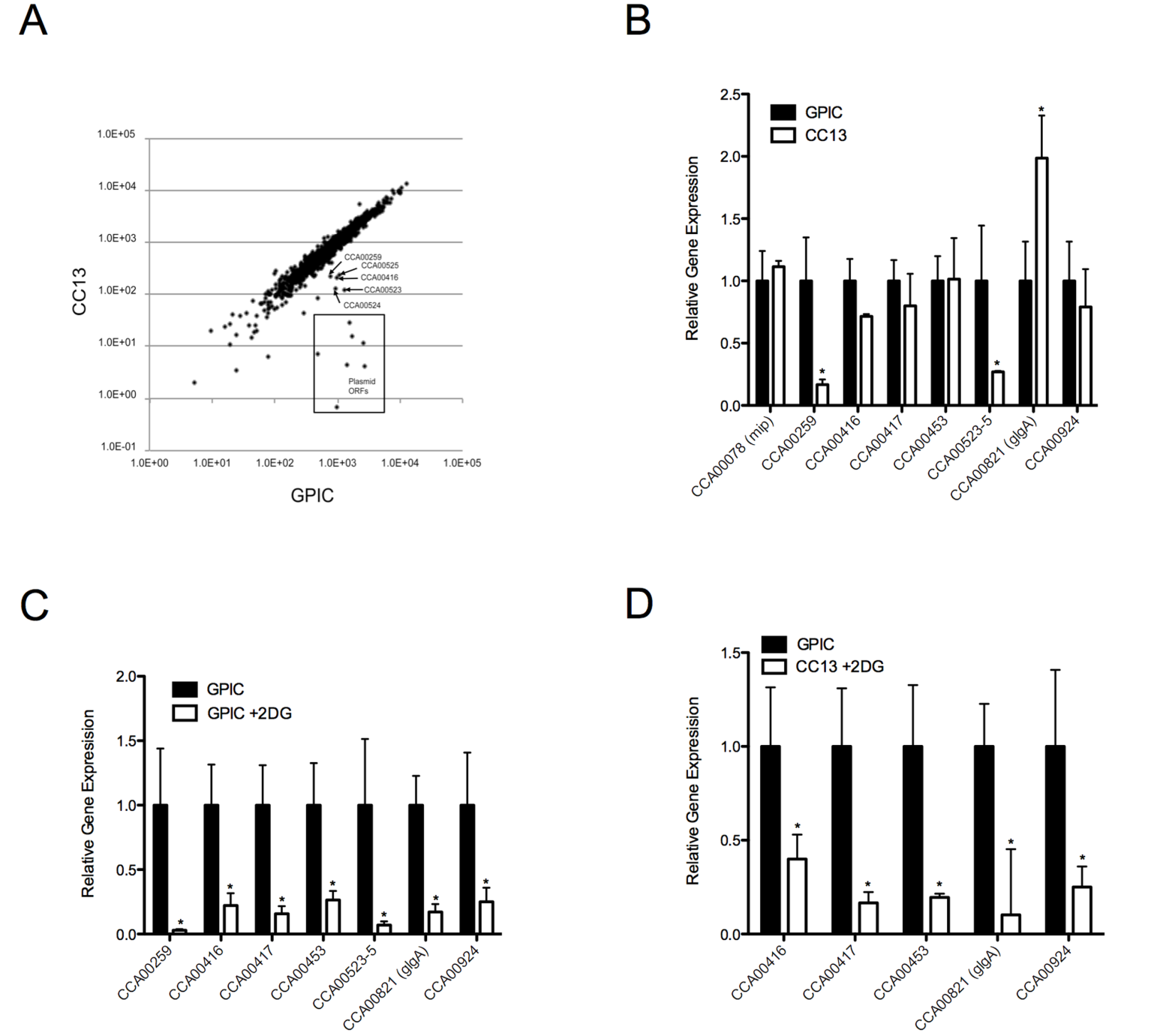


Scatter plot illustration of microarray comparison of the transcriptional profile of *C. caviae* GPIC and its plasmid-cured derivative CC13 30 hours after infection.

Supplemental Fig. 2.
